# A mixed fixed ratio/progressive ratio procedure reveals an apathy phenotype in the BAC HD and the z_Q175 KI mouse models of Huntington’s disease

**DOI:** 10.1371/4f972cffe82c0

**Published:** 2012-04-25

**Authors:** Stephen Oakeshott, Russell Port, Jane Cummins-Sutphen, Jason Berger, Judy Watson-Johnson, Sylvie Ramboz, Neil Paterson, Seung Kwak, David Howland, Dani Brunner

**Affiliations:** Principal Scientist at PsychoGenics, Inc.; Research Associate at PsychoGenics, Inc.; Research Associate at PsychoGenics, Inc.; Research Associate at PsychoGenics, Inc.; Breeding Manager at PsychoGenics, Inc.; Senior Director, Neurodegenerative Disorders at PsychoGenics, Inc.; Sr. Principal Scientist, Cognition, PsychoGenics, Inc.; Director of Target Biology; Director of In Vivo Biology, CHDI Foundation Inc, Princeton, NJ; Senior VP Behavioral R&D at PsychoGenics, Inc.

## Abstract

Apathy, characterized by generally reduced interest in and likelihood to perform goal-directed actions, is a recognized symptom of Huntington’s disease (HD), a devastating neurological disorder caused by a CAG repeat expansion of the Htt gene located on chromosome 4. The present experiments used a modified progressive ratio task that incorporated a fixed-ratio schedule of reinforcement component to assess consummatory behavior, and a progressive-ratio schedule component that required increasing numbers of lever-presses for successive reinforcers (0.01 ml of evaporated milk). The studies revealed an apathetic phenotype in two mouse models of HD, with decreased response rates either overall or only at higher ratio requirements in the progressive-ratio component relative to wild-type controls. Based on the procedure used (within-session fixed- and progressive-ratio components), it is proposed that an observed phenotype can be ascribed either specifically to reduced motivation to work for food reinforcement or more generally to deficits in consummatory behavior. This procedure provides a simple means to assess this type of phenotype in rodents, with issues in consummatory vs. incentive motivation reflected in general alterations in fixed- versus progressive alterations on an escalating-ratio schedules respectively, providing translational measures of the amotivation/apathy construct of the human realm to the homologous construct of incentive motivation in preclinical models of human disease.

## Introduction

Huntington’s disease (HD) is an autosomal dominant genetic disorder, resulting from expansion of a stretch of CAG repeats near the N-terminus of the Htt HD gene on chromosome 4, coding for the protein huntingtin. At present, HD is invariably fatal, with the interval from the presentation of initial symptoms to death typically being around 15 to 20 years 1. Clinically, HD is characterized by a triad of symptom groups, with progressive dysfunction of motor, psychiatric and cognitive performance, with the non-motor aspects of the disease generally held to cause a significant burden to patients and caregivers even relatively early in disease progression 2.

Apathy, frequently defined as a lack of motivation characterized by diminished goal-oriented behavior, is a major symptom in HD, as well as other major neuropsychiatric disorders (for review, see 3). In patients, motivational problems are seen once disease state is relatively advanced, being observed in over half of moderately and severely affected patients 4. Lack of motivation is reflected in both lack of pleasure in normally rewarding activities and diminished behavior aimed at obtaining such rewards. Although very prevalent in all neurodegenerative disorders and a major contributor to both the psychiatric pain of the patient and the burden for the caregiver, apathy has rarely been the focus of in-depth research in the clinical domain, due to lack of consensus about its definition and proper measurement with traditional scales like SANS, PANSS and UPDRS. Now that specific and reliable scales, such as Marin’s apathy evaluation scale (AES), have been set up and validated 5 6 7, the scene is appropriately set for advancing the understanding of this symptom in both neurodegenerative and psychiatric disorders. Consummatory (direct pleasure derived from the reward, or hedonia) and incentive-anticipatory components (that drive reward- or goal-directed behavior) of motivation have been recognized both in the clinic and in the experimental analysis of behavior 8 9 10 11.

Historically, researchers have utilized simple fixed-ratio schedules of reinforcement (unchanging response requirement, typically at a low effort level) or even simpler dose-response preference of rewards such as sucrose to measure consummatory behaviors 12. By contrast, progressive-ratio schedules (increasing number of responses are required to obtain consecutive deliveries of the reinforcer 13), have been used to provide a more reliable and unambiguous measure of incentive motivation 12 13, in addition to providing a measure of reinforcer magnitude/efficacy. Typically, the final response requirement completed in the test session is referred to as the ‘break-point’ and has been used as the single performance measure. Recently however, some have proposed the use of response rates at specific response ratios as a useful and informative output measure 14 although there is a risk of potential confounds with motor deficits if appropriate controls are not included in the experimental design. Satiation is a potential confounding factor in this type of study, although the problem is much reduced under PR versus FR schedules since delivery of reinforcers is typically far more limited under PR because of increasing response requirements.

The motivation for the present experiments is to explore operant schedules of reinforcement to define an homologous behavioral endpoint of the apathy symptom in the mouse, which, coupled with the relatively large number of preclinical HD models now available 15 16 17 18 19, would be of great value for screening of possible therapeutics. Therefore, a mixed low effort fixed- and escalating- effort progressive-ratio (FR and PR, respectively) procedure was utilized to explore consummatory behavior (relevant to immediate rewarding or reinforcing properties of a stimulus with the FR component), and incentive motivation (PR component). Two mouse models of HD were characterized in the task; specifically, the BAC HD full-length transgenic model generated by William Yang 15, and the z_Q175 knock-in model generated at PsychoGenics Inc. 19. As far as these authors are aware, neither mouse has been shown to exhibit an apathetic phenotype. It was predicted that an HD mouse model with an apathetic phenotype will reduce its work rate earlier in the test session, at a lower ratio requirement, than will a healthy WT.

## Experiment 1A

The initial experiments were conducted using adult BAC HD mice, a full-length transgenic model developed in the laboratory of X. William Yang 15

Materials and Methods

Subjects

WT and transgenic BAC mice (BAC HD, CHDI-007-(3)(1)) were generated crossing dams carrying ~97 stable CAGCAA repeats on a C57Bl/6J with FVB/NJ male mice at the Jackson Laboratory (Bar Harbor, ME) and shipped to the test facility as adults. Mice were maintained in opti-MICE cages (Animal Care Systems, CO) on a 12:12 light cycle with free access to water.

Once acclimated to our colony, the animals were pair-housed and food restricted, with WT mice reduced to 85% of their free-feeding body weights. The BAC Tg mice, which develop excess fat deposits, were food-restricted gradually to the point where a matched control group consumed comparable amounts of food to 85% WT animals in a 30-min free feeding test, at which point they were slightly heavier than controls (mean 36.5 g for BAC mice, 28.1 g for WT controls on the initial day of training). Mice were maintained at their new target weights by daily provision of limited quantities of food (BIO-SERV 500mg pellets).

A single group of female mice was evaluated, 8 animals per genotype. The mice had previously been evaluated in the PhenoCube (described in 20), an automated phenotyping system, but were naïve to operant boxes and had never before been food restricted. All mice were 73 weeks old ± 1 week on the initial day of instrumental training and were 79 weeks old ± 1 week on the initial day of mixed FR5/PR training.

All studies were carried out in strict accordance with the recommendations in the Guide for the Care and Use of Laboratory Animals, NRC 2011. The protocol was approved by the Institutional Animal Care and Use Committee of Psychogenics, Inc. (PHS OLAW animal welfare assurance number A4471-01), an AAALAC International accredited institution (Unit #001213).

Equipment

Mice were tested in standard mouse operant chambers (Med Associates, VT) with the floor area measuring 8.5" long x 7.0" wide and 5.0" high walls. Each chamber contained a nosepoke recess, which could be illuminated by a small embedded bulb, located centrally on the wall opposite the food magazine, though these were not employed in this testing. The boxes also contained two retractable levers, one on either side of the food magazine. The chambers were located within individual sound attenuating shells, with a fan mounted at one end of the sound-attenuating cubicle which was active throughout. Reinforcement was provided by time-limited access to 0.01 ml of evaporated milk (Carnation™) delivered via a dipper. The hardware was controlled and all events were recorded by the Med-PC IV software package.

Behavioral procedures - pretraining

Following food restriction and magazine training, all animals were trained to lever press via a simple free operant procedure, where a single lever was inserted throughout a 40 min session and lever pressing was reinforced with 4-s access to an evaporated milk reinforcer on a response-initiated fixed-interval 20 sec (FI20) schedule. No reinforcement was delivered without a lever press. Animals were trained to a criterion, requiring them to obtain 50 reinforcers across 2 consecutive sessions. Training was carried out daily Monday to Friday, with the animals resting over the weekends, and continued until all test mice had reached the criterion. Following criterion achievement, the animals were switched to an FR5 reinforcement schedule and trained for a further 3 sessions, with the sessions lasting either for 40 min or until the animal earned 75 reinforcers, whichever occurred soonest.

Behavioral procedures - PR testing

*Simple PR. *Following completion of this training, the animals were switched to PR training, with the number of responses required to earn a reward escalating steeply as the session progressed. The ratio sequence employed in these studies required animals to make the following number of responses for each consecutive reinforcer: 1, 5, 10, 15, 20, 30, 40, 50, 60, 80, 100, 120, 140, 180, 220, 260, 300, 380, 460, 540, 620, 780, 940, 1100, 1260, 1580, 1900. These sessions continued either until the animals made no responses for at least 5 min, or until the session had continued for 90 min. This training continued for 26 sessions.

*Mixed FR5/PR.* Finally, the animals were switched to a mixed FR5/PR schedule, designed to measure their responding on a low-effort (the FR5) and an escalating effort (the PR) schedule within a given test session. These sessions opened with 10 min of training on the FR5 schedule, with no reinforcer cap, then continued for a further 80 min with the same PR schedule as previously. This testing continued for 24 sessions.

During FR5/PR training in Experiment 1A, the animals inadvertently received an extra session of FR5-alone training between sessions 7 and 8 due to experimenter error. Data from this session were discarded.

Statistical methods

Data were evaluated via repeated measures analysis carried out with SAS (SAS Institute Inc.) using Mixed Effect Models, based on likelihood estimation. The models were fitted using the procedure PROC MIXED [21]. Genotype and one of Session, Reinforcer Bin or Response Requirement, depending on the particular dataset, and their interactions were considered in all the models, with sex evaluated separately where appropriate. Significant interactions were followed up with simple main effects analyses. Analysis of pretraining and response rate data was carried out with t-tests or one-way ANOVAs using Statview (SAS Institute Inc.). An effect was considered significant if p < 0.05.

Power analysis was performed using PASS11© version 11.0.5 from NCSS, LLC, using Repeated Measures ANOVA (proc. #166). The goal was to find the minimum number of subjects per group required to obtain a power = 0.80 for a significant statistical effect for several potential therapeutic effect sizes.

Behavioral results

*Pretraining*. All animals in this study readily acquired the lever press response, passing the reinforcer criterion in the initial two days of testing. The majority of the mice obtained the maximum 75 reinforcers in each session of FR5 training, so no analysis was attempted of their overall number of responses. There were no significant changes in response rates across the three test sessions or differences between the two genotypes.

*Simple PR. *Data from the progressive ratio only test sessions (26 sessions) is presented in the left panel of Figure 1, below, and clearly indicates that the BAC HD mice (filled symbols) earned fewer reinforcers than did WT controls (open symbols) throughout training (Genotype main effect: F(1,13) = 46.2, *p < 0.0001*; Session main effect: F(25,350) = 3.11,* p < 0.0001*; Trend towards a Genotype x Session interaction: F(25,350) = 1.50, *p < 0.06; *Figure 1).

**Number of reinforcers earned during progressive ratio training across sessions in Experiments 1A (left panel) and 1B (right panel):**
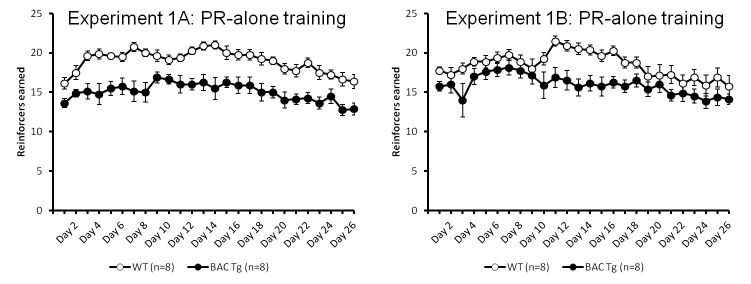

*Mixed FR5/PR.* While the data from this PR training are of clear interest, in the absence of clearly defined breakpoints in the animals’ responding the possibility remains that these slower overall response rates in the BAC Tg mice might be related to some motor impairment. Accordingly, data from the final mixed FR5/PR phase of this experiment is presented in the left panels of Figure 2, split into low effort FR5 training (upper panel) and escalating effort PR training (lower panel). In the FR5 phase, both groups showed similar numbers of reinforcements earned across sessions. In contrast, as in the PR-alone training phase (see above), the BAC Tg mice earned significantly fewer reinforcers than WT controls during the PR component (Genotype main effect: F(1,14) = 25.0, *p < 0.001). *Both groups earned fewer reinforcers in the PR phase as testing progressed, independently of genotype (Session main effect: F(23,322) = 3.29, *p < 0.0001*).

**Summarized data from mixed FR5/PR training across sessions in Experiment 1A (left panels) and Experiment 1B (right panels):**
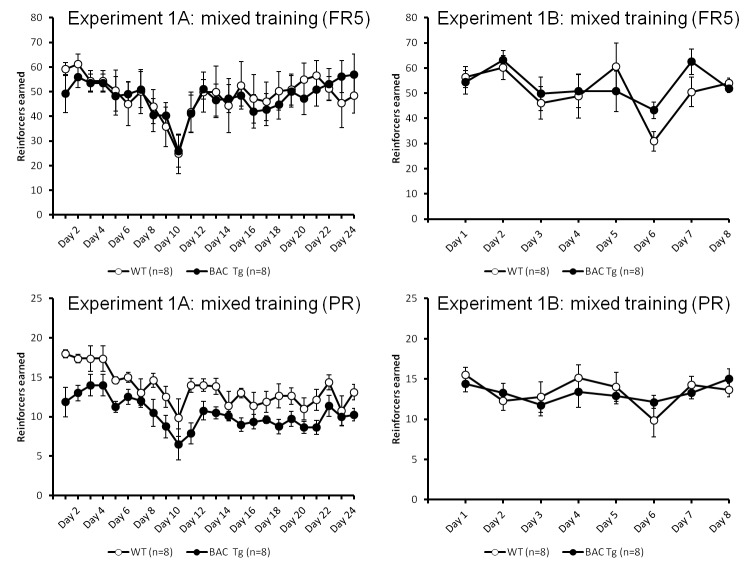
Data from the 10 min FR5 training period are presented in the upper panels, while data from the 80 min PR training period are presented in the lower panels.

Finally, to break down these overall difference by the specific response requirements, response rates were calculated per reinforcer earned within each session, with these data then collapsed across training sessions for each mouse. In Experiment 1A, these response rate data were only collected during the final 8 sessions of FR5/PR training and are presented in the left panels ofFigure 3 below, with data from the FR5 component in the upper panel and from the PR component in the lower panel. Since not all animals earned the same number of total reinforcers in each phase, these data are truncated at the point where most mice were still contributing to the dataset, at 30 reinforcers for the FR5 phase and 12 reinforcers for the PR phase. No differences were apparent in the rate of responding per reinforcer in the FR5 phase, with all animals working at a comparable level. Both groups responded more slowly as session time elapsed independently of genotype (Session main effect: F(39,541) = 8.40, *p < 0.0001*). Similarly, early in the PR component where the level of effort required per reinforcer was low, both groups of mice appeared comparable (Figure 3, lower left panel). However, as the level of effort increased, the BAC Tg mice responded less than did WT controls, with this effect recorded starting at the ratio 50, although the differences missed significance at the ratio 100 (Response Ratio main effect and Response Ratio x Genotype interaction: smaller F(10,137) = 2.15, *ps < 0.05;* simple main effects: smallest F(1,137) = 3.98, all *ps < 0.05*).

**Collapsed response rate data from the final 8 sessions of FR5/PR training broken down by reinforcer for Experiment 1A (left panels) and Experiment 1B (right panels):**
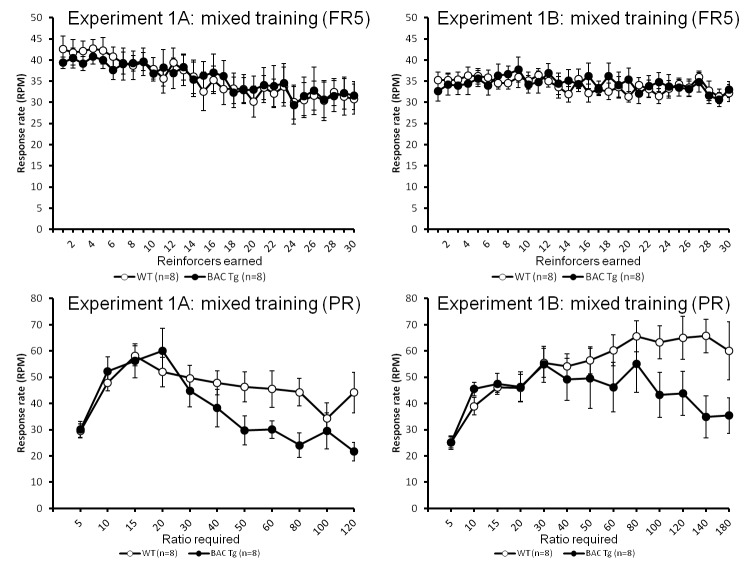
Data from the FR5 training period (upper panels) are labeled per reinforcer earned, while those from the PR training period (lower panels) are instead labeled by the ratio requirement, such that each datapoint indicates the mean response rate while working on that ratio.

## Experiment 1B

Subjects

A second cohort of BAC HD (CHDI-007-(3)(1)) female mice were evaluated at an earlier age, both to confirm the phenotype was robust and to potentially extend the finding to an earlier timepoint. These animals were experimentally naïve and were housed and maintained as described in Experiment 1A, except that they were maintained at their target weights by daily access to an unlimited quantity of food for a 2 hour period following completion of testing, rather than by daily provision of a limited quantity of food. These animals were 46 weeks old ± 1 week on the initial day of instrumental training and were 55 weeks old ± 1 week on the initial day of mixed FR5/PR training.

Equipment and procedures

All equipment and behavioral procedures were as described in Experiment 1A, except that these animals received 5 sessions of FR5 training following acquisition of the lever press and only 8 sessions of mixed FR5/PR training at the end of the study.

Behavioral results

*Pretraining. *As in Experiment 1A, all animals in this study readily acquired the lever press response, reaching the reinforcer criterion in the initial two days of testing. In the FR5 training phase, both groups responded faster as training progressed independently of genotype (Session main effect: F(4,56) = 25.7, p < 0.0001).

*Simple PR. *Overall session data from the PR-alone training phase, presented in the right panel ofFigure 1 above, again clearly indicated that the BAC Tg mice earned fewer reinforcers than WT controls, with no major effects of training(Genotype main effect: F(1,14) = 10.2, *p < 0.01*; Figure 1).

*Mixed FR5/PR.* The number of reinforcers earned in the 8 sessions of mixed FR5/PR sessions is summarized in the right panels of Figure 2, with the FR5 period presented in the upper panel and the PR period presented in the lower panel. Both groups earned similar number of reinforcers in the FR5 components although they earned fewer reinforcers as training progressed and showed some variability during the last few sessions, with BAC Tg mice earning more reinforcers on day 6 (Genotype x Session interaction and Session main effect: smaller F(7,98) = 2.30, *ps < 0.05;* simple main effects analysis: F(1,98) = 5.11, *p < 0.05, *Figure 2 upper panel)*. *Similarly, in the PR component of this experiment, both groups earned slightly less reinforcers as sessions progressed but this effect was independent of genotype (Session main effect: F(7,98) = 3.59*, p < 0.01; *Figure 2, lower right panel).

Finally, analysis of the response rate as a function of the number of reinforcers earned was carried out. As in the previous experiment, these data were analyzed only for the period during the sessions where the majority of animals contributed data, namely the first 30 reinforcers in the FR5 phase and 14 reinforcers in the PR phase (up to the 180 ratio). These data show that the response rate in the FR5 component declined with successive reinforcers independently of genotype (Reinforcer Bin main effect: F(39,546) = 4.96, *p < 0.0001; *Figure 3, upper right panel). Analysis of the PR period showed that responding increased with the ratio requirement and suggested a drop in the BAC Tg mice with higher ratios, although the genotype difference missed significance (Genotype x Response Requirement interaction: F(12,167) = 1.49, p < 0.14; Response Requirement main effect: F(12,167) = 13.8, p < 0.0001).

## Experiment 2A

Testing was also carried out in the z_Q175 KI mouse, a novel knock-in model derived at Psychogenics from the CAG140 KI mouse (see 18), now carrying between 180 and 195 CAG repeats (see 19 for detailed description of this novel line), to study incentive motivation in a second HD model.

Subjects

WT, homozygous and heterozygous (het) knock-in (KI) mice were bred in our facility by crossing pairs of z_Q175 KI (CHDI-015-1) het mice, on a congenic C57Bl/6J background. A small cohort of female z_Q175 KI mice was evaluated in this study, with 5 mice tested per genotype. Homozygous mice were also included in the initial cohort but failed to acquire the lever press response in a timely fashion and were accordingly dropped from the study. One WT animal became sick following completion of instrumental pretraining and was subsequently dropped from the experiment.

The mice were maintained throughout in opti-MICE cages (Animal Care Systems, CO) on a 12:12 light cycle with free access to water. Prior to this testing, these animals underwent a comprehensive behavioral test battery but were naïve to operant boxes and had not previously been food restricted. These mice were single housed in opti-MICE cages prior to the start of this testing, with all animals reduced to 85% of their *ad libitum* body weight by daily feeding of limited quantities of food (BIO-SERV 500mg pellets) throughout the test period.

All mice were 74 weeks old ± 1 week on the initial day of instrumental training and 77 ½ ± 1-week old on the initial day of mixed FR5/PR training. The z_Q175 het mice in this experiment had a mean CAG repeat length of 193 repeats, ranging from 189 to 196 repeats.

Equipment and procedures

All equipment and behavioral procedures were as described in Experiment 1A. This experiment was conducted over a shorter timeframe than were Experiments 1A and 1B, with the animals receiving 4 sessions of FR5 training following acquisition of the lever press, 6 sessions of PR-alone training and finishing with 8 sessions of mixed FR5/PR training at the end of the study.

Behavioral results

*Pretraining.* WT and het mice reached the reinforcer criterion after between 2 and 6 days with no genotype differences. Evaluation of the response rates in the four FR5 training sessions (data not shown) showed that both groups responded faster over the course of training independently of genotype (Session main effect: F(3,21) = 16.5, *p < 0.0001).*

*Simple PR. *Day-by-day data from the 6 PR-alone training days showed that WT mice earned more reinforcers as training progressed, whereas the z_Q175 mice were quite stable. Both groups performed similarly at the end of training (Session x Genotype interaction: F(5,35) = 2.98, *p < 0.05*, Session effect in WT: F(5,35) = 6.32, *p < 0.001, *Figure 4, left panel).

**Number of reinforcers earned during progressive ratio training across sessions in Experiments 2A (left panel) and 2B (right panel):**
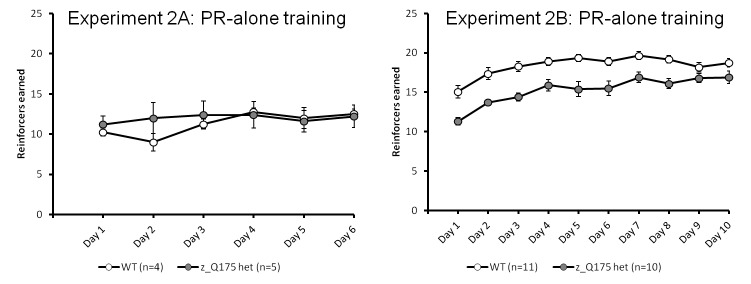


*Mixed FR5/PR.* During the PR component both groups showed variable performance across days but without a consistent effect of either genotype or training session (Session main effect: F(7,49) = 2.75, *p < 0.05, *Figure 5, upper left panel). Analysis of the PR data revealed that both groups earned less reinforcers as training progressed independently of genotype (Session main effect: F(7,49) = 7.70, *p < 0.001*; Figure 5, lower left panel).

**Summarized data from mixed FR5/PR training across sessions in Experiment 2A (left panels) and Experiment 2B (right panels):**
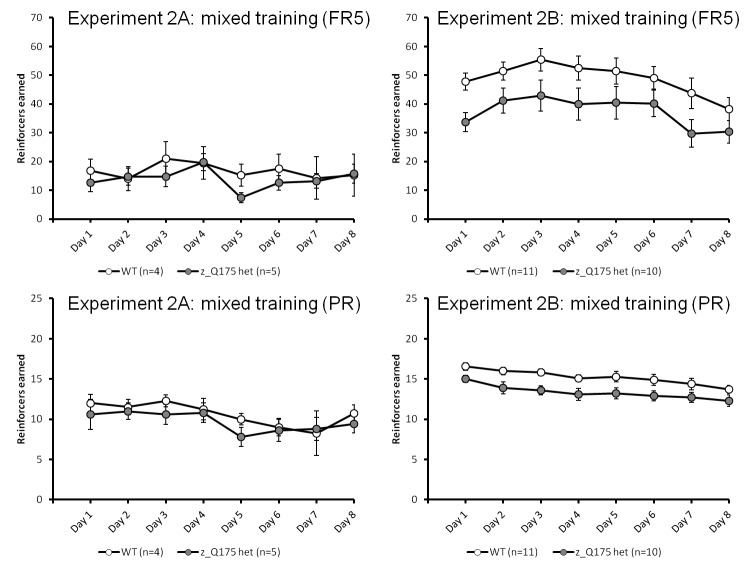
Data from the initial FR5 training period are presented in the upper panels, while data from the PR training period are presented in the lower panels.

Analysis of the FR5 response rate as a function of the number of reinforcers earned (for the initial 15 reinforcers) showed that the z_Q175 het mice responded consistently less than WT mice (Genotype main effect: F(1,7) = 7.68, *p < 0.05; *Figure 6 upper left panel*)*.

The inverse U-shaped response rate curve in the PR component (truncated at the 120-response requirement) suggested that z_Q175 het mice responded slightly less than WT mice although this difference did not reach significance (Genotype main effect: F(1,7) = 3.74, *p < 0.10; *Response Requirement: F(10,62) =5.11, *p < 0.001;* Figure 6 bottom left panel).

**Collapsed response rate data from the 8 sessions of FR5/PR training broken down by reinforcer for Experiment 2A (left panels) and Experiment 2B (right panels):**
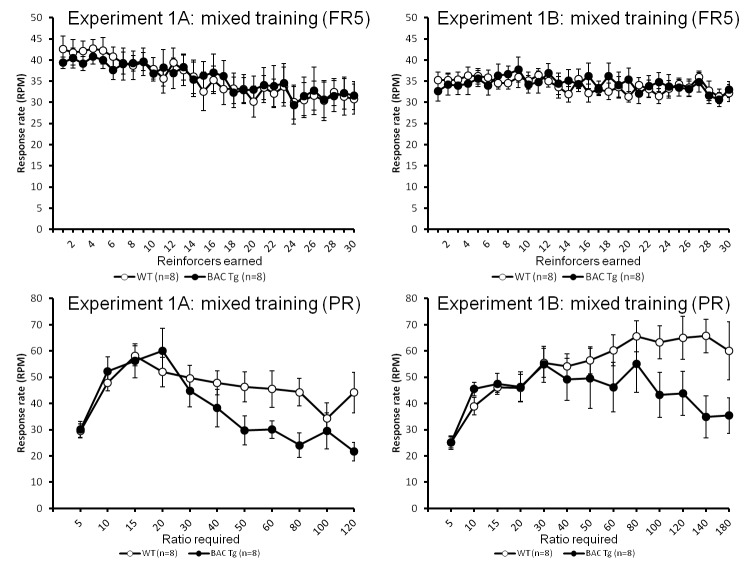
Data from the FR5 training period (upper panels) are labeled per reinforcer earned, while those from the PR training period (lower panels) are instead labeled by the ratio requirement, such that each datapoint indicates the mean response rate while working on that ratio.

## Experiment 2B

As response rates in Experiment 2A were low overall regardless of genotype, likely due to the advanced age of the mice, a second group of z_Q175 KI mice were investigated at an earlier timepoint, to establish if a more robust phenotype might be detected at this age.

Subjects

A mixed sex cohort of het and WT knock-in mice, 11 per genotype, was bred in our facility by crossing z_Q175 (CHDI-15-1) het male animals with WT females, all on a congenic C57Bl/6J background. The mice were housed and maintained as described for Experiment 2A, above. Prior to this testing, these animals had been evaluated in the PhenoCube, a comprehensive behavioral screening platform, and the tapered beam motor assay, but were naïve to operant boxes and had never before been food restricted. The mean CAG repeat length in the z_Q175 het mice in this experiment was 189 repeats, with the cohort ranging from 184 to 201 repeats. One female z_Q175 het animal became sick during the final phase of testing (FR5/PR testing) and was unable to continue. As a precaution, all data from this animal was excluded from the dataset analyzed. These animals were 27 weeks old ± 1 week on the initial day of instrumental training and 30 ± 1 weeks old on the initial day of mixed FR5/PR training.

Equipment and procedures

All equipment and behavioral procedures were as described in Experiment 1A, except that the maximum length of both the PR-alone and the FR5/PR test sessions was reduced to 60 min. The FR5/PR sessions still began with 10 min of the FR5 schedule, now followed by 50 min of PR training. Animals in this experiment received 5 sessions of FR5 training following acquisition of the lever press, followed by 10 sessions of PR-alone training and concluding with 8 sessions of the mixed FR5/PR procedure.

Behavioral Results

*Pretraining.* All mice acquired the lever press within two days with the exception of one female z_Q175 het animal, which required 6 days. While WT mice increased their response rate as training progressed, responding in the z_Q175 mice responding was lower on all days and did not change with training (Genotype x Session interaction: F(4,67) = 2.66, *p < 0.05*; simple main effects: smallest F(1,67)= 5.17, all *ps < 0.05*; Session effect for the WT: F(4,67) = 10.5, *p < 0.0001*; not shown).

*Simple PR. *z_Q175 het mice earned fewer reinforcers across the PR training sessions than did WT controls, although both groups earned more as training progressed (Genotype main effect: F(1,17) = 38.0*, p < 0.0001;* Session main effect: F(9,153) = 13.7, *p < 0.0001; *Figure 4, right panel)*. *Female mice earned fewer reinforcers (Sex main effect: F(1,17) = 11.3, *p < 0.01; *not shown).

*Mixed FR5/PR.* During the FR5 component of the FR5/PR phase, the total number of reinforcers earned in both groups increased and then decreased across sessions, z_Q175 het mice earning fewer reinforcers than did WT mice overall (Genotype main effect: F(1,17) = 4.75, *p < 0.05*; Session main effect: F(7,119) = 8.27, *p < 0.0001;* **Figure 5, upper right panel).

In the PR component the same pattern was shown across sessions, with fewer reinforcers earned with more training and reduced responding in the mutants (Genotype main effect: F(1,17) = 10.2,*p < 0.01; *Session main effect: F(7,119) = 14.7, *p < 0.001; *Figure 5, bottom right panel*). *Female mice earned fewer reinforcers than did males (Sex main effect: F(1,17) = 4.58, *p < 0.05; *not shown).

During the FR5 period, the overall response rate for both groups was lower within sessions as more reinforcers were earned, with the z_Q175 het mice responding significantly less for almost all reinforcers (Genotype x Reinforcer Bin interaction: F(29,493) = 3.31, *p < 0.0001*. Simple main effect analysis for reinforcers 1-14, 16-17, 19: smallest F(1,493) = 5.58, all *ps < 0.02;* Figure 6, upper right panel). There were no sex effects.

Finally, response rate as a function of response requirement within sessions in the PR component of the FR5/PR phase, as in Experiment 2A, showed an inverse U-shaped response function for both groups with significantly lower overall responding in the z_Q175 mice but no significant interaction with the required level of effort (Genotype main effect: F(1,19) = 9.89, *p < 0.01*; Response Requirement main effect: F(10,192) = 9.92, *p < 0.0001*; Figure 6 lower right panel). There were no sex effects.

## Power Analyses

In order to evaluate the utility of this procedure for screening work, power analysis was carried out on the Genotype main effect and the Genotype x Response Requirement interaction in data from the PR period of the final FR5/PR period of each of the four experiments. The output of these analyses is summarized in Table 1 below, indicating that the assay has reasonable power in both lines tested. In the BAC HD mice, around 35 mice per group are required for a 50% effect size in the Genotype x Response Requirement interaction. For the z_Q175 het animals, around 36 mice per condition are required, again for a 50% effect size but here with the better power seen in the overall Genotype effect, reflecting the more global phenotype seen in this line.



Table 1: Required n/condition for 80% power with the effect sizes specified.

## Discussion

These experiments indicate age- and genotype-dependent apathy or incentive motivation deficits in a mixed fixed/progressive-ratio (FR/PR) schedule of reinforcement in two mouse models of HD, the BAC HD, a full-length transgenic model generated by William Yang 15, and the z_Q175 KI model derived at Psychogenics 19 from the CAG140 KI model originated in the laboratory of Dr. Zeitlin 18. Specifically, the BAC Tg mice exhibit deficits in the number of rewards obtained during PR training at both ages tested compared to their WT controls. However, under the mixed FR/PR schedule, the older BAC Tg mice exhibited decreased reinforcers earned, and decreased response rates at high ratio requirements in the PR component, with no observed differences from WT controls in the FR component. By contrast, the younger BAC Tg mice exhibited only a trend (p < 0.08) toward a significant decrease in response rate in the PR schedule. Thus, with increasing age, BAC HD mice exhibit the emergence of a selective deficit in progressive-ratio versus fixed-ratio responding for a potent reinforcer (evaporated milk), when data are expressed either as reinforcers earned or response rate at specific response ratios. By contrast, the younger z_Q175 KI mice appear to reveal a more severe phenotype than do the BAC HD mice, with moderately decreased performance at all phases of testing (FR and PR schedules), whether data were expressed as reinforcers earned or response rate per ratio, compared to WT controls. Moreover, homozygous mice from this line were not able to acquire the basic lever pressing response. In preliminary data from a small test cohort (n=4-5), older z_Q175 KI appeared to exhibit a less obvious phenotype. A significant effect of genotype was observed only for the response rate in the FR component of the mixed FR/PR test procedure, possibly due to the reduced level of responding in the WT controls (i.e. a ceiling effect). Based on this pattern of results, it may be that the aged BAC Tg mice exhibit a selective deficit in motivation that is due to incentive motivation (less goal directed behavior or less “wanting”) rather than hedonic motivation (less pleasure derived from reward, or less “liking”). Young z_Q175 KI mice may exhibit a more profound deficit, involving both incentive and hedonic motivation, resulting in both FR and PR deficits. Older z_Q175 KI and WT controls exhibited much lower levels of responding overall, reducing the opportunity to assess genotype effects on motivation.

It is generally the case that these mice respond slightly more rapidly in the intermediate phase of the PR (requiring ~30 responses per reinforcer) than during the FR5 periods, raising the possibility that these data could be influenced by some motor issue preventing the HD mice from matching WT controls at these ratios. In Experiments 1A and 1B, it is clear from inspection of these data that the underlying cause of the genotype differences in these experiments is related to a decline in response rate in the HD animals as the ratios increase, rather than simply to a failure to match increased responding in the WTs. In conjunction with the apparent ability of the BAC Tg mice to maintain their responding during the FR5 periods, it does not appear that a simple motor phenotype could be responsible for these results. In the z_Q175 KI mice tested in Experiment 2B, it similarly does not appear that simple motor deficits could explain these results. Although the z_Q175 mice responded more slowly than WT controls at all times, they are still able to raise their response rate to ~40 RPM during the early part of the progressive ratio, suggesting that their relatively slow responding in the FR5 period cannot be simply ascribed to an inability to respond faster; more generally, as z_Q175 around 26 weeks of age show only mild hypoactivity 19, it is reasonable to assume that motor deficits are not confounding this assessment of motivational state. Therefore, it is most likely that the observed deficits in responding in these experiments do indeed reflect the pattern of motivation abnormalities outlined above, with only the aged z_Q175 KI mice (and their WT control subjects) appearing to exhibit a motor deficit in terms of decreased maximum response rate under both schedules of reinforcement, compared to all other mouse strains/ages.

Typically, progressive ratio studies in the literature report performance in terms of 'break points', recording the ratio requirement at which the animals stop responding for a period of time as a measure of the maximum effort the subjects will impart 13. In the present studies, likely as a consequence of the training procedure employed, the test animals failed to exhibit clear breakpoints within the allowed intervals, continuing to make intermittent responses for long periods of time. Consequently, the approach described here was implemented instead, measuring their work-rate per ratio requirement.

As has been described elsewhere 22, operant tasks generally offer a very valuable tool for assessment of transgenic animals generally and HD mouse models specifically, providing a reasonably high throughput, largely automated and sensitive set of tools to assess these animals. This procedure, requiring only a lever press and a food magazine, is ideally suited to preclinical screening in any facility with a simple operant setup and will ideally provide a helpful platform for preclinical screening to ameliorate or prevent the emergence of motivation issues in HD patients.

In summary, the present data provide evidence for age- and genotype-dependent apathy/amotivational deficits in a mixed fixed-/progressive-ratio (FR/PR) schedule of reinforcement in two mouse models of HD, the BAC HD full-length transgenic model generated by William Yang 15 and the z_Q175 KI model 19. These data extend the range of HD characteristics currently modeled in preclinical subjects, and may provide a valuable screening tool for novel HD therapeutics.

## Competing Interest Statement

CHDI Foundation provides financial support for PLoS Currents: Huntington Disease. Editorial responsibility for all content remains entirely within the remit of the Public Library of Science, the Editors, and Board of Reviewers.

Stephen Oakeshott, Russell Port, Judy Watson-Johnson, Jason Berger, Jane Sutphen, Neil E. Paterson, Sylvie Ramboz and Dani Brunner are or were all employed by PsychoGenics, Inc., a for-profit institution.

The authors have declared that no further competing interests exist.

## References

[1] Roos RA, Hermans J, Vegter-van der Vlis M, van Ommen GJ, Bruyn GW (1993) Duration of illness in Huntington's disease is not related to age at onset. J Neurol Neurosurg Psychiatry 56: 98-100. 10.1136/jnnp.56.1.98 PMC1014774 8429330

[2] Craufurd D, Snowden J (2002) Neuropsychological and neuropsychiatric aspects of Huntington’s Disease In: Bates G, Harper PS, Jones L, editors. Huntington's Disease. Oxford: Oxford University Press. pp. 62-94.

[3] Ishizaki J, Mimura M (2011) Dysthymia and apathy: diagnosis and treatment. Depress Res Treat 2011: 893905. 10.1155/2011/893905 PMC3130974 21747995

[4] Caine ED, Shoulson I (1983) Psychiatric syndromes in Huntington's disease. Am J Psychiatry 140: 728-733. 10.1176/ajp.140.6.728 6221669

[5] Marin RS, Biedrzycki RC, Firinciogullari S (1991) Reliability and validity of the apathy evaluation scale. Psychiatry Research 38: 143-162. 10.1016/0165-1781(91)90040-v 1754629

[6] Marin RS (1991) Apathy: a neuropsychiatric syndrome. J Neuropsychiatry Clin Neurosci 3: 243-254. 10.1176/jnp.3.3.243 1821241

[7] Clarke DE, Ko JY, Kuhl EA, van Reekum R, Salvador R, et al. (2011) Are the available apathy measures reliable and valid? A review of the psychometric evidence. J Psychosom Res 70: 73-97. 10.1016/j.jpsychores.2010.01.012 PMC3902773 21193104

[8] Barbano MF, Cador M (2007) Opioids for hedonic experience and dopamine to get ready for it. Psychopharmacology (Berl) 191: 497-506. 10.1007/s00213-006-0521-1 17031710

[9] Gard DE, Kring AM, Gard MG, Horan WP, Green MF (2007) Anhedonia in schizophrenia: distinctions between anticipatory and consummatory pleasure. Schizophr Res 93: 253-260. 10.1016/j.schres.2007.03.008 PMC1986826 17490858

[10] Treadway MT, Zald DH (2011) Reconsidering anhedonia in depression: lessons from translational neuroscience. Neurosci Biobehav Rev 35: 537-555. 10.1016/j.neubiorev.2010.06.006 PMC3005986 20603146

[11] Bindra D (1978) How adaptive behavior is produced: a perceptual-motivational alternative to response reinforcements. Behavioral and Brain Sciences 1: 41-52.

[12] Arnold JM, Roberts DC (1997) A critique of fixed and progressive ratio schedules used to examine the neural substrates of drug reinforcement. Pharmacol Biochem Behav 57: 441-447. 10.1016/s0091-3057(96)00445-5 9218268

[13] Hodos W (1961) Progressive ratio as a measure of reward strength. Science 134: 943-944. 10.1126/science.134.3483.943 13714876

[14] Killeen PR, Posadas-Sanchez D, Johansen EB, Thrailkill EA (2009) Progressive ratio schedules of reinforcement. J Exp Psychol Anim Behav Process 35: 35-50. 10.1037/a0012497 PMC2806234 19159161

[15] Gray M, Shirasaki DI, Cepeda C, Andre VM, Wilburn B, et al. (2008) Full-length human mutant huntingtin with a stable polyglutamine repeat can elicit progressive and selective neuropathogenesis in BACHD mice. J Neurosci 28: 6182-6195. 10.1523/JNEUROSCI.0857-08.2008 PMC2630800 18550760

[16] Hodgson JG, Agopyan N, Gutekunst CA, Leavitt BR, LePiane F, et al. (1999) A YAC mouse model for Huntington's disease with full-length mutant huntingtin, cytoplasmic toxicity, and selective striatal neurodegeneration. Neuron 23: 181-192. 10.1016/s0896-6273(00)80764-3 10402204

[17] Mangiarini L, Sathasivam K, Seller M, Cozens B, Harper A, et al. (1996) Exon 1 of the HD gene with an expanded CAG repeat is sufficient to cause a progressive neurological phenotype in transgenic mice. Cell 87: 493-506. 10.1016/s0092-8674(00)81369-0 8898202

[18] Menalled LB, Sison JD, Dragatsis I, Zeitlin S, Chesselet MF (2003) Time course of early motor and neuropathological anomalies in a knock-in mouse model of Huntington's disease with 140 CAG repeats. J Comp Neurol 465: 11-26. 10.1002/cne.10776 12926013

[19] Menalled L, Kudwa A, Miller S, Fitzpatrick J, Watson-Johnson J, et al. (in prep.) Comprehensive behavioral characterization of a new knock in mouse model of Huntington’s disease. 10.1371/journal.pone.0049838 PMC3527464 23284626

[20] Oakeshott S, Balci F, Filippov I, Murphy C, Port R, et al. (2011) Circadian Abnormalities in Motor Activity in a BAC Transgenic Mouse Model of Huntington’s Disease. PLoS Curr. 2011 April 5; 3: RRN1225. 10.1371/currents.RRN1225 PMC3072044 21479110

[21] Singer J (1998) Using SAS PROC MIXED to Fit Multilevel Models, Hierarchical Models, and Individual Growth Models. Journal of Educational and Behavioral Statistics 23: 323.

[22] Trueman RC, Dunnett SB, Brooks SP (2011) Operant-based instrumental learning for analysis of genetically modified models of Huntington's disease. Brain Res Bull. 10.1016/j.brainresbull.2011.03.015 21440048

